# Systems thinking in practice when implementing a national policy program for the improvement of women's healthcare

**DOI:** 10.3389/fpubh.2023.957653

**Published:** 2023-09-29

**Authors:** Monica E. Nyström, Sara Tolf, Vibeke Sparring, Helena Strehlenert

**Affiliations:** ^1^Department of Learning, Informatics, Management and Ethics, Medical Management Centre, Karolinska Institute, Stockholm, Sweden; ^2^Department of Epidemiology and Global Health, Umeå University, Umeå, Sweden; ^3^Centre for Health Economics, Informatics and Health Services Research, Stockholm Health Care Services, Stockholm, Sweden; ^4^Stockholm Gerontology Research Centre Foundation, Stockholm, Sweden

**Keywords:** systems thinking, healthcare improvement, policy implementation, public health, healthcare services

## Abstract

**Introduction:**

Interest in applying systems thinking (ST) in public health and healthcare improvement has increased in the past decade, but its practical use is still unclear. ST has been found useful in addressing the complexity and dynamics of organizations and welfare systems during periods of change. Exploring how ST is used in practice in national policy programs addressing complex and ill-structured problems can increase the knowledge of the use and eventually the usefulness of ST during complex changes. In ST, a multi-level approach is suggested to coordinate interventions over individual, organizational, and community levels, but most attempts to operationalize ST focus on the individual level. This study aimed to investigate how ST is expressed in policy programs addressing wicked problems and describe the specific action strategies used in practice in a national program in Sweden, using a new conceptual framework comprising ST principles on the organizational level as an analytical tool. The program addresses several challenges and aims to achieve systems change within women's healthcare.

**Methods:**

The case study used a rich set of qualitative, longitudinal data on individual, group, and organizational levels, collected during the implementation of the program. Deductive content analysis provided narrative descriptions of how the ST principles were expressed in actions, based on interviews, observations, and archival data.

**Results:**

The results showed that the program management team used various strategies and activities corresponding to organizational level ST. The team convened numerous types of actors and used collaborative approaches and many different information sources in striving to create a joint and holistic understanding of the program and its context. Visualization tools and adaptive approaches were used to support regional contact persons and staff in their development work. Efforts were made to identify high-leverage solutions to problems influencing the quality and coordination of care before, during, and after childbirth, solutions adaptable to regional conditions.

**Discussion/conclusions:**

The organizational level ST framework was useful for identifying ST in practice in the policy program, but to increase further understanding of how ST is applied within policy programs, we suggest a multi-dimensional model to identify ST on several levels.

## 1. Introduction

Many public health and social issues are complex and so are the interventions that can affect them. Such multi-dimensional issues often represent ‘wicked problems', i.e., problems that involve multiple sectors, multiple organizational levels, and many actors, and that are dynamic and difficult to define (1–4). This complexity makes it difficult to implement, evaluate, and scale up health interventions (5). How wicked problems should be addressed has been debated as most of the ill-structured and wicked problems defy solutions (6, 7). Usually, they are addressed as if they could be solved, or by reducing them into well-structured problems to control them. An alternative could be to use a coping strategy that focuses on the process of repeatedly trying to resolve the wicked problem (8). Soft-law initiatives, i.e., non-legislative modes of policymaking based on voluntary cooperation, have been a way to deal with such complex policy problems, especially in the Nordic countries (9). However, the focus on the process, the aim to incorporate multiple and competing perspectives on the problem, and the continuously changing contextual conditions make it difficult to lead such soft-law initiatives.

An approach based on systems thinking (ST) can be useful for tackling complex issues when leading soft-law initiatives (2, 10). ST has also been suggested as an aid when identifying high-leverage solutions that can improve multiple health outcomes (11). The interest in applying ST in public health and healthcare improvement has increased in the past decade (12–14). Even so, relatively few applied studies focus on ST within public health, and more research is needed to understand how ST is used in this field (15–17).

Systems thinking is a theoretical approach found to be useful in addressing the complexity and dynamics of organizations and welfare systems when trying to change a current situation (2, 10, 11, 13, 18). ST has multiple origins from diverse scientific traditions, and it involves a wide range of terminologies, theories, and tools (10). Unlike reductionist approaches, ST considers the complexity of a phenomenon and its context, e.g., that interventions are interdependent on each other and on the environment (19–21). A recent review shows that most articles published on ST are conceptual (17). Thus, there is a need for more knowledge about how ST can be put into action within public health and healthcare improvement (18), and the need for further studies and development of practical applications is highly relevant (10, 17).

There are challenges in studying how ST is manifested in practice. At the same time, identifying how ST can be expressed in actions and strategies is an important step in building knowledge about the practical application of ST in public health (22) and the mechanisms behind the effects of quality improvement initiatives (23). ST emphasized the coordination of interventions across multiple levels of change, e.g., individual, organizational, and community levels (24). This “multi-level” approach is in line with what is needed when national policy programs address ill-structured or wicked problems in public health and healthcare.

Most attempts to operationalize and measure ST focus on the *individual level* relating ST to individuals' understandings, abilities, skills, and cognitive processes. Studies on ST emphasize individuals' knowledge and abilities, for example, to be able to understand how the system is organized, managed, and led; to understand and be able to manage system stakeholders and networks; and to have the ability to conceptualize, model, and understand dynamic change (24–26) as important to facilitate change. To assess ST on the individual level, system attributes have been used when investigating and comparing ST preferences with preferences for reductionism (27–29). There are also attempts made to define and measure ST as a cognitive process (30). Richmond's taxonomy of “thinking skills” (31, 32) has been used in several studies [e.g., (33, 34)], where, for example, more complex ST skills have been linked to better decision-making (33). Measurement of ST on the individual level mostly relies on the subjective judgment of one's experiences or preferences, sometimes in relation to described fictive situations.

Implementation of policy programs typically involves many different types of actors, and, usually, there is a team responsible for the program, which potentially can benefit from ST to address wicked problems and the dynamic changes inherent in them. Some indications of the use of ST on a *group level* have been described in the literature. Different people have different objectives and perspectives, which affects the situation at hand (35, 36). Addressing a complex and problematic situation requires understanding multiple perspectives, and Soft Systems Methodology is one ST approach designed to tackle diffuse real-world problems (37). Mental models of managerial teams' ST have been related to organizational learning processes, especially when the teams' shared understandings and action strategies change (38). More recently, factors that foster collaborative ST in teams have been studied (39). Studies of ST at the group level focus on a mixed social and cognitive process. Concepts described in other research fields, such as shared cognition [e.g., (40)], team mental modeling [e.g., (41)], sense-making as a social process [e.g., (42, 43)], and team learning (44), can aid the understanding of the use of ST in groups. Finding ways to achieve shared cognition and team mental models among key actors involved in policy programs is important to achieve systems change (45, 46).

Operationalization of ST *on the organizational level* is also scarce. Indicators that can provide insights into how and to what extent organizations apply ST are limited or even seen as lacking, especially within the public health domain (22, 47). Smith et al. (47) have recently proposed a framework for ST in public health, which combines ST, collaborative inquiry and action, and systemic science and methods. The framework is based on previous public health frameworks (48), and the framework's initial concepts (49) were further refined drawing on insights from public health scientists and practitioners with experiences from nine policy programs (22). It has been further operationalized and tested by Wilkins et al. (22), and eight principles of a systems orientation have been proposed (Table 1). Wilkins et al. (22) also developed and tested quantitative indicators of the ST principles within organizations (i.e., state public health departments) focusing on the area of state injury and violence prevention. Their attempt is focused on evaluation and is considered a first step “toward measuring ST at the organizational level in public health” [23, p.76]. Their study provides quantitative indications of an ST aspect in terms of numbers, presence or absence, or percentage, e.g., Convene partners—the number of internal (health departments) and external partners engaged to advance injury and violence prevention activities/strategies/programs/policies per year. It is proposed that such indicators can be used to identify ST in an organization. However, it does not provide a detailed description of how ST is used in practice or describe strategies that can aid those who work with soft-law initiatives addressing ill-structured or wicked problems.

**Table 1:** Definitions of the eight principles of ST on the organizational level (22).

**ST principles**	**Definitions**
P1. Convene partners	Bringing together partners to (1) identify gaps and needs, (2) identify assumptions, (3) identify high-leverage points, (4) identify high-leverage solutions, (5) evaluate the process, and (6) disseminate data. Partners should include those who have diverse content expertise and expertise across multiple roles; reflect the unique attributes, culture, and characteristics of the community; have decision-making power; and are likely to bring a divergent perspective. This also includes intentional strategies for engaging partners (such as identifying common ground) and for strengthening the quality of partnerships (such as building trust and improving communication).
P2. Seek understanding	Gathering information from the community to better understand challenges, learn about community culture, and identify strengths. This includes acquiring and assessing various sources of data and evidence that are relevant to the context and the questions being asked. It also includes identifying what contributes to community challenges, how these contributing factors relate to one another, and how making changes to these contributing factors may influence health (and other) outcomes, and/or potentially lead to unintended consequences.
P3. Surface assumptions	Identifying partners' and stakeholders' “mental models” or assumptions about the community, its challenges, and the solutions needed to improve its health. This process also includes identifying gaps between different mental models held by various partners.
P4. Reflect and learn	Continually reviewing emerging information, identified assumptions, and lessons learned to collaboratively develop and refine a shared vision for improving community outcomes. This includes creating environments in which people are encouraged and supported to regularly reflect and learn from emerging findings, and to contribute to practice-based research.
P5. Find leverage	Identifying solutions, innovations, and public health actions that are likely to be appropriate for the needs of the community, efficient, high impact, and sustainable. This includes solutions that (1) are based on data and have demonstrated impact in similar communities, (2) address “upstream” factors and social determinants that contribute to community challenges, (3) are uniquely tailored and combined to have the most impact in the local context, (4) galvanize broad support and coordination among partners, and (5) support efficiency and sustainability by improving public health infrastructure.
P6. Manage resources	Leveraging and coordinating existing resources, such as funding and staff, to support and sustain collective action. This includes cross-training or co-locating staff to facilitate coordinated activities and braiding funding streams to adequately and sustainably support them.
P7. Respond rapidly	Alongside collaborative partners, taking action and continuously improving solutions as issues, data, and lessons learned emerge. This includes discontinuing strategies that are unsuccessful, amplifying those that are working, catalyzing action among partners and stakeholders, addressing unintended consequences, and re-evaluating priorities when needed.
P8. Translate findings	Synthesizing and sharing relevant findings, data, and information with partners, stakeholders, and the public. This includes engaging partners and key stakeholders in the process of determining which findings and information are important to share, and the best ways of disseminating and packaging that information.

This study focuses on how ST was used in practice within a national soft-law initiative that addressed several wicked problems and was launched in a decentralized healthcare system. To find indications of if and how ST is used in practice within such policy program, observations of individual skills and social and cognitive processes in groups would benefit from being complemented with other indications (22), and Wilkins et al.'s ST principles have a potential to enrich our understanding of how ST reveals itself in practical activities and the action strategies used within a policy program.

This study aimed to investigate how ST is expressed in practice in complex policy programs addressing wicked problems and describe the specific action strategies used in practice in a national program in Sweden, using a new conceptual framework comprising ST principles on the organizational level (22) as an analytical tool. Providing narrative descriptions of how ST is used in practice, complementary to Wilkins et al.'s (22) test of indicators, can aid others involved in similar soft-law initiatives and policy programs. The underlying assumption behind the study is, in line with previous research, that ST can facilitate change and development within public health [e.g., (10, 13, 14, 16, 49)], by promoting a more holistic understanding of complex social phenomena in complex settings and by supporting collaborative approaches to address ill-structured problems.

## 2. Materials and methods

This explorative case study uses a rich set of longitudinal data collected during the multi-year implementation of a national policy program in Sweden. The program was chosen partly due to convenience (i.e., access to data) but mainly due to its complexity, representing a comprehensive policy program aimed at several large improvement areas representing wicked problems within a large, complex national setting comprising many geographical areas (i.e., 21 self-governed regions), types of care providers (primary and specialized hospital care, and public and private providers), types of care (e.g., delivery care and neonatal care), units (e.g., primary healthcare units and delivery care clinics), and actors. The study was reviewed by the Regional Ethical Review Board in Stockholm, and they found a formal ethical approval was not needed (ref no. 2018/620-31).

### 2.1. Empirical setting—the Swedish healthcare system

The Swedish healthcare system is comparatively decentralized and divided into 21 regional self-governing authorities and 290 municipalities. The regions, which vary in size and demography, are responsible for the provision of healthcare services, and the municipalities for providing home healthcare and social care. The Swedish Association of Local Authorities and Regions (SALAR) is a member organization representing the self-governing regions and municipalities and, as such, is an influential policy actor. Healthcare is mainly tax-funded, and most care providers are publicly owned. Maternal healthcare is provided at outpatient maternal healthcare clinics led by midwives (50). These clinics work with health in connection to pregnancy, support to families, contraceptive counseling, and public health. During pregnancy, women have access to free controls starting from weeks 8 to 12. Unless there is a health problem, women do not see a doctor during the pregnancy. After pregnancy, routine post-partum care is offered (50).

Improving Women's Health and Care before, during, and after Pregnancy program (WHCP program) aims to affect an extensive system, i.e., maternity care, antenatal care, delivery care, post-partum care, and, from 2018, neonatal care, in all 21 regions. The organizations of these subsystems have regional variations. Maternity can be part of the same subsystem as delivery care and gynecology or be organized under primary healthcare. The variation also concerns the number of private care providers, mainly offering maternity care before childbirth. Private care providers were essentially absent in some regions and more common in the large urban regions.

### 2.2. Characteristics of the national policy program

The program was initiated in late 2015 to be implemented between 2016 and 2019. It is based on agreements between the national and regional political levels, i.e., between the Ministry of Health and Social Affairs and SALAR, the latter a national organization representing the 21 self-governed regions that attend to, support, and coordinate the regions' common interests. Instead of addressing the complex challenges and (wicked) problems via laws or regulations, they were addressed by an agreement that the regions would put efforts into improving certain areas, based on and adapted to the local situation, and receive funding for this from the government. The agreements were based on mutual trust rather than on control or enforcement. The first agreement was followed by several additional agreements, increasing the scope of the program, and extending the implementation period until the end of 2023. Thus, the implementation of the policy program stretches over almost 9 years. The program aims to improve women's sexual and reproductive health and maternity, antenatal, and post-partum care. The agreement is more decentralized than some previous ones [e.g., (51–53)] where the funding was linked to performance measures.

**Figure 1:**
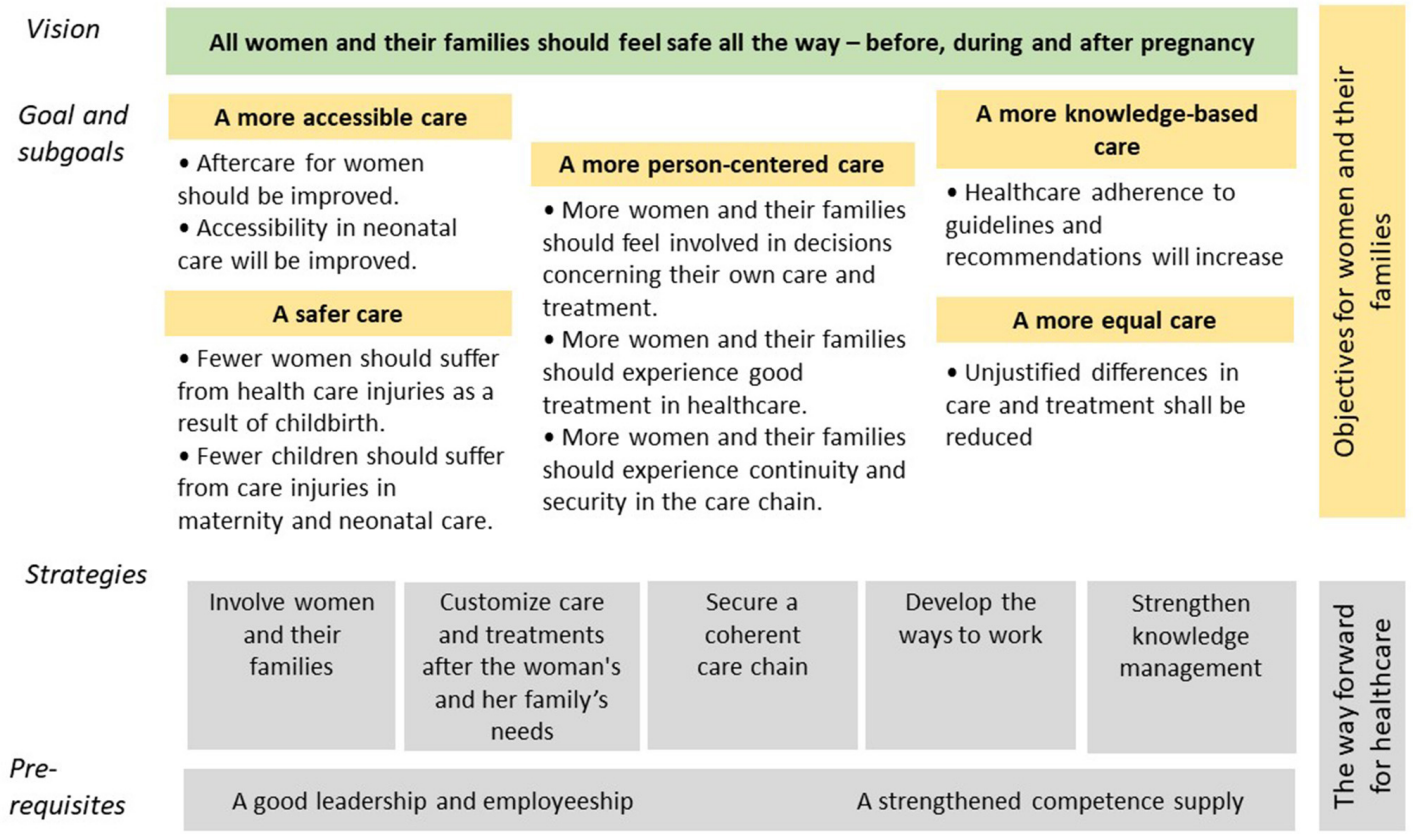
Strategic plan of the WHCP program.

In 2015, a national program team was formed at SALAR, responsible for leading, coordinating, supporting, and following up on the program's progress and its outcomes. This team had little mandate to enforce the program and did not influence the allocation of the program's finances, which were sent directly to the regions. In 2018, the national program team developed a strategic plan based on the agreement, see Figure 1—adapted from (54), which formed the basis for the forthcoming program. The strategic plan visualized and described the program vision, goals, prerequisites, and overarching strategies.

The program is decentralized, implying that the 21 regions are responsible for identifying needs, prioritizing, and implementing interventions to improve their work within the strategic areas of the program. Regional contact persons, appointed by the director of health in each region, function as the nodes for contact and interaction with the WHCP program team at SALAR. The funding was distributed directly to the regions based on the size of their population, with a smaller amount designated to the program team, and the funding for some special missions was given to public authorities, e.g., the National Board of Health and Welfare (NBHW). Thus, the regions could decide how to distribute the funding to reach the goals of the program, based on their knowledge of the regional and local conditions. The Swedish Agency for Health and Care Services Analysis was given the mission to evaluate the program's outcomes.

### 2.3. Data collection

Since 2017, data about the program have been collected and compiled in a comprehensive case study database by external researchers (among them authors MEN, ST, and VS), as a part of a longitudinal (still ongoing) research project. The database consists of semi-structured individual and group interviews conducted with program team members (2018–2021), contact persons from all regions (2018 and 2020–2021), and external program evaluators (2020); non-participant observations of meetings (2017–2022); and documents (e.g., reports, evaluations, policy documents, meeting agendas, and presentation material), survey data, quality registry data, and national and regional publicly available statistics.

In this study, we have used a representative sample of interviews, observations, and archival data sources chosen to represent various types of data, content, actors, and time periods from the database which cover a 5-year period (March 2017 to March 2022), excluding outcome data, i.e., quality registries (Table 2). The sample consisted of 12 interviews with the program team (2018; 2020), 4 representative interviews with regional contact persons, 20 observations of meetings and conferences (2017–2022), and 34 documents (2016–2022). Interviews with the program team and with contact persons covered similar themes: national or regional program organization; strategies and activities; conditions and enabling and hindering factors; communication; support; follow-up and evaluation; effects; learnings; and plans for the next year. In two rounds of interviews, the program team members described their experiences of situations, important activities, perceived effects, and, if they can, the intentions and rationales behind them. Interviews contain both current and retrospective data. Interview guides can be found in Supplementary material 1–4. Interviews with regional contact persons were added to represent their experiences of the program activities and action strategies. Twenty non-participant observations of activities performed within the program and their detailed content (i.e., what was presented and discussed) were collected. The observation template and an example of observation data can be found in Supplementary material 5. Descriptions of activities and their content could be found in documentation, i.e., archival data (see Supplementary material 5 for examples of different types).

**Table 2:** WHCP program case database, from which data were selected and analyzed in the study.

**2015-11-01 to 2022-12-31 data sources 2017-03 - 2022-03**	**Specifications**	**Data**	**Data analyzed in this study**
Interviews (*n =* 58)	- National program team	−2018 (*n =* 6); 2020 (*n =* 6) tot 12	−12 interviews
	- Regional contact persons	−2018 (*n =* 23) 2020 (*n =* 22) tot 45	−4 interviews
	- Evaluators	−2021 (*n =* 1)	
Observations (*n =* 54)	- Program team meetings	−2017-2022-02 (*n =* 27)	−10 observations
	- Contact person meetings 2–4 h	−2017-2022-02 (*n =* 15)	−8 observations
	- Contact person conferences 4x2 days	−2017–2020 (*n =* 8)	−2 observations (of 2 conferences x 2 days)
	- National meetings/workshops-−1 day	−2017–2019 (*n =* 1)	
	- Regional meetings 6 h-−1,5 day	−2017–2020 (*n =* 6)	
Archival data (*n =* 263)	- Agendas for the above meetings	−2017–2022 (*n =* 61)	−25 documents
	- PowerPoint presentations from meetings	−2017–2022 (*n =* 73)	−9 documents
	- Reports and web reports—SALAR	−2017–2022 (*n =* 17)	
	- Reports—Evaluators	−2017–2022 (*n =* 2)	
	- The 21 region's yearly activity reports	−2017–2022 (5x21=105)	
	- Other documents	−2017–2022 (*n =* 15)	

### 2.4. Data analysis

The definitions of the eight ST principles (see Table 1) in the refined conceptual framework (22) were used to identify and categorize indications of the practical use of ST within the program.

First, the researchers familiarized themselves with the eight ST principles by discussing examples of what type of program content and data potentially could contain indications of the principles (Table 1). Then, relevant data sources, representative of the program process over time, were identified and selected from the large database (Table 2).

An iterative approach based on deductive content analysis (55) was applied using the definitions of the principles in the framework (22), presented in Table 1. Multiple data sources (e.g., interviews and archival data) were used to triangulate information about activities and expressed strategies fitting the definition of each principle. The first step of the analysis was performed by two researchers (ST and MN) by coding data information in the data sample using the principles in the framework. After sharing these extracts, all four researchers met in six 1–3 h-long meeting sessions to scrutinize and further discuss the interpretation of the identified text in relation to each principle and to reach a consensus on program findings that could represent the principles, if found. The procedure intended to ensure reliability and validity in the interpretations of the qualitative data and resulted in a few alterations of the narrative descriptions used (i.e., one activity description was not used, and one was placed under another ST principle). Interview quotations and extracts of text used for the illustration of the principles were chosen during this process. Finally, a synthesis of data on each principle, including identified action strategies, formed the basis for a narrative description of how ST was used in the program.

## 3. Results

In this section, narrative descriptions are presented about how the organizational level ST principles (P1–8), put forward by Wilkins et al. (22), were applied in practice in the WHCP program. For each principle, examples from different data sources can be found in Supplementary material 5, while Tables 3–10 provide the action strategies and detailed examples for each principle.

**Table 3:** Principle 1—convene partners expressed in practice in the WHCP program.

**Principle**	**Action strategies**	**Detailed description**
*P1 convene partners*	Engage a multi-professional program team	Team members were deliberately chosen by the team leader to represent a variety of professional expertise and experience from different parts and levels of the healthcare system (e.g., public health, maternity care, communication, and HR).
	Create a network of regional contact persons	A network of appointed contact persons in each region was established. Forums for interaction were mainly via group meetings (e.g., regional conferences, dialogue tours visiting all regions, digital and face-to-face meetings, and workshops and program-specific web-based collaborative platforms). This partnership was initially used to clarify expectations and enhance interactions across system levels but developed over time to involve all ST principles.
	Interact with national authorities involved in similar issues	The team interacted with actors from several national authorities, e.g., NBHW, The Swedish Public Health Authority, and key actors, such as the national healthcare IT platform and representatives from the Ministry of Health and Social Affairs.
	Collaborate with program teams leading other national policy agreements	The team collaborated with other national policy agreements in related areas such as the agreements on developing a national structure for knowledge management in healthcare; improving available and person-centered primary care; and improving the situation for women subjected to physical, psychological, or sexual violence.
	Involve external program evaluators and academic researchers	The program team engaged with the external program evaluators and a group of academic researchers following the program and invited them to participate in and contribute to program activities.
	Interact with existing social networks	Some networks were readily available and hosted by SALAR, (e.g., networks of regional politicians, healthcare directors, and HR directors). Others were networks of representatives from professional organizations (e.g., the Swedish Association of Midwives) or actors with specific functions (e.g., the national network of midwives with a coordination function in their organization). These arenas were mainly used to spread information and to get input and feedback on planned or performed activities.

### 3.1. Principle 1—convene partners

The principle Convene Partners concerns identifying, reaching, involving, and engaging the right actors at the right time and comprises both the variety of involved stakeholders and partners and what they do together, which includes the forthcoming principles. How to identify, reach, and involve the right actors at the right time depends on the complexity of the program and its setting.

**Figure 2:**
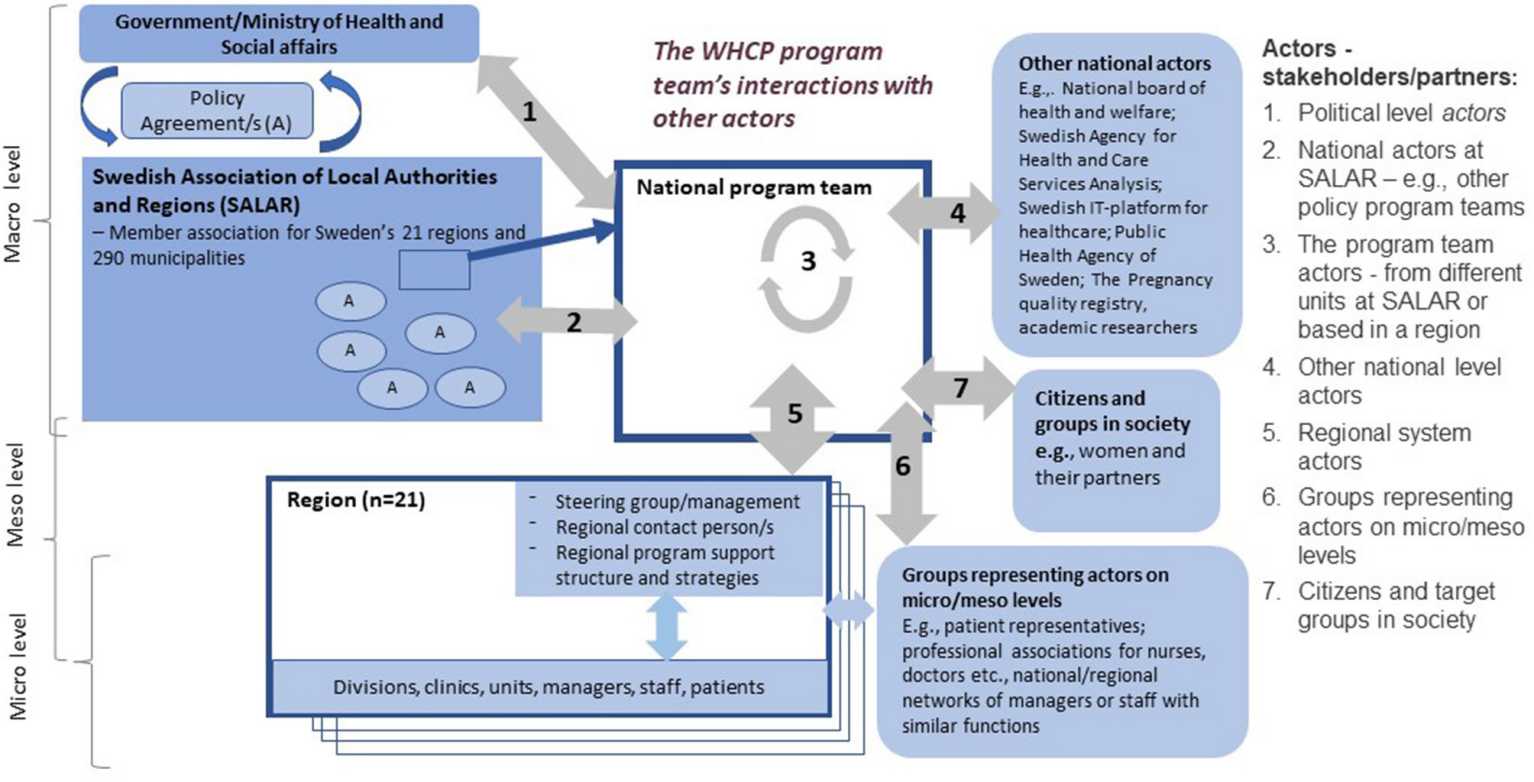
Overview of the types of actors involved in the national program.

To address the issues and reach the WHCP program goals of a more equal, accessible, safe, knowledge-based, and person-centered care for women over the entire country meant identifying and involving many different actors and professions from different parts and levels of the healthcare system, from politicians to patient representatives. This was recognized by the program team members and described in interviews as being important from the start of the program. It was also visible in the amount and type of actors involved in the various program activities over time (see Figure 2). Different actors were involved in the identification and **analyses** of challenges, problems, and contextual influences and in problem-solving, planning, and follow-up activities, either regularly or for limited periods of time. This ensured that many perspectives could be considered when planning and implementing program activities. The regular interaction with other national authorities and programs was perceived by the program team members to reduce the risk of launching competing activities. The interaction with and consideration of the different actors and their interests require significant amounts of time and skills on behalf of the program team. Reaching and involving higher regional decision-makers was difficult. They were informed when the program was initiated and later in their monthly national meetings. Depending on the chosen regional contact person and regional strategy meant that key actors on a higher regional level could have been more or less involved in the realization of the program intentions. The team coordinating the program at the national level was based at SALAR, which is a members' and an employers' organization for all the regions in Sweden. This created unique opportunities for the team to get a national overview and facilitate linkages between national and regional levels. This platform secured a mandate to facilitate collaborations and coordinate ongoing system changes. Due to the decentralized approach regarding regional power over the choices of problem areas and interventions, the regional contact persons were key actors in stimulating regional change. The five main action strategies identified are described in Table 3. Figure 2 provides an overview of the types of actors involved in the program and the action strategies used.

### 3.2. Principle 2—seek understanding

The principle Seek Understanding concerns gathering information from the context to better understand what contributes to challenges and strengths, how these contributions relate to each other, and how making changes to these contributions may influence outcomes and potentially lead to unintended consequences.

The WHCP program comprised several multi-faceted issues, e.g., equity in care, attracting and keeping competent staff, patient safety, availability of care, person-centered care, and integrated care. How to understand this range of issues, what contributes to the challenges and also consider regional variations and context-specific conditions for providing healthcare, was addressed in meetings and some team members expressed in interviews as a challenge.

The analyses of problems and needs were an important strategy on behalf of the program team. Mappings and gap analyses were conducted and presented in reports and then communicated, discussed, and reflected on during several meetings with regional actors. Monitoring different media also became important for understanding the region's various conditions and challenges. The program team's efforts to gather information to better understand regional and contextual challenges, often together with program stakeholders and partners, were perceived to contribute to a better understanding of both the system features and the complex improvement areas the program aimed to affect, especially for new members in the program team and contact persons, and also for others involved, for example, from national authorities. The strategic plan developed in 2018 (Figure 1) was an important tool for aiding the understanding of the program, especially as each region could, based on their context-specific needs, choose which areas of the program to focus on and which interventions to use. The seven main action strategies identified are described in Table 4.

**Table 4:** Principle 2—Seek Understanding expressed in practice in the WHCP program.

**Principle**	**Action strategies**	**Detailed description**
*P2 seek understanding*	Perform problem analyses, needs assessments, knowledge reviews, gap analyses, and review existing solutions	Seventeen public reports were produced in 7 years, addressing initial and emerging problems and needs, identifying gaps, and discussing various ways to address them. The initial report on the status of women's sexual and reproductive health and healthcare services in 2016 identified areas in need of improvement. A deeper analysis followed in 2018 complemented by a report identifying several ways to improve the identified challenges. The reports were perceived by contact persons as supportive of their regional work.
		Arrange arenas with regional representatives to collaboratively seek an understanding of the program issues in varied settings	To manage and understand the complexity the team developed program activities, such as network meetings and workshops with regional contact persons and other regional representatives with a mix of competencies and professions, to analyze program issues in detail and in variable contexts.
		Perform dialogue tours to each region to discuss the regional situation	Yearly visits initiated in 2018 covered meetings with decision-makers and representatives from various parts of women's healthcare (e.g., maternity care, antenatal care, delivery care, post-partum care, and neonatal care) and provided complementary information on regional conditions and needs and perspectives on the strategic areas. The wide range of issues addressed by the program was discussed with all regions in 2018. Regional differences in how care was organized and functioned and in perceived problems to support care providers and implement the program were revealed.
		Develop and visualize a strategic plan of the program areas, goals, and strategies to aid understanding of the program	Early in the program, regional representatives asked for clarification on what was expected of the regions. A clarified and visualized strategic plan of the main parts and strategies of the program was developed by the program team in 2018 and presented in the report ‘Strategies for women's health'. Constructing and visualizing the strategic plan was an attempt to clarify the overarching goals and describe the program logic and the general strategies to achieve the goals. The team used the plan in meetings with regional representatives and in the dialogue tours.
		If information is lacking for developing ways to gather information on women's experiences	In the focus area of person-centered care and the strategy to involve women and their partners, available information on the women's experiences of care before, during, and after childbirth was scarce. Therefore, the development of a National Pregnancy Survey was initiated. In 2021, the first results from this survey were presented in a report.
		Monitor, engage, and discuss activities of related national policy programs and projects with stakeholders to build a holistic and mutual understanding	The team regularly monitored the activities of national stakeholders, e.g., NBHW, the Swedish Agency for Health Technology Assessment and Assessment of Social Service, the Swedish Food Agency (breastfeeding), and the Public Health Agency of Sweden. Stakeholders from related programs and government missions (e.g., authorities developing new clinical practice guidelines) were invited to discuss their work together with the contact persons, and opportunities for mutual understanding were provided. Concurrently, the team let stakeholders know what was happening in the WHCP program. Interrelations among ongoing national policy programs at SALAR were highlighted and discussed in meetings and persons working in other programs in nearby areas were engaged in the program team, e.g., by part-time employment.
		Monitor information presented in media on the situation in the regions	Delivery care was of high interest to the media during the period and media reports had an impact locally and regionally and on the program team's work on the national level. The team's communication officer monitored media reports, more intensively from 2021 and onwards after the launch of the National Pregnancy Survey and a growing concern about the increasing shortage of midwives in many regions. During this period, media reports were discussed in program team and contact person meetings and on the program's IT platform.

### 3.3. Principle 3—surface assumptions

The principle Surface Assumption concerns identifying partners' and stakeholders' assumptions about the focus areas and the program and the challenges and solutions needed to improve care and women's health. The overarching goals and structure of the program were set in negotiation between actors representing the government, politicians, and decision-makers from the regions and representatives from SALAR. Thus, the agreement was originally based on the mental models and assumptions of those involved in negotiating, writing, and signing the agreement, mirroring mainly a political perspective on issues and on what constitutes good care for women before, during, and after pregnancy. The negotiations resulted in a high degree of freedom regarding the implementation of the agreement, and the goals were rather general to suit stakeholders with divergent needs (see Figure 1). Due to the program's comprehensive character and being a national initiative aiming to influence processes in the autonomous regions, the program team would need to identify the underlying assumptions held by actors on multiple system levels, which could reduce confusions and conflicts, and facilitate the program team's choices of implementation support.

The program team worked to surface assumptions held by stakeholders directly involved in the implementation, and those held by the contracting parties in the policy agreement, i.e., the government and SALAR. This is partly expressed in Principle 2 in the ways the team tried to seek understanding by involving different actors, but it was not explicitly described in the team members' interviews as a strategy. For program team members and contact persons, the knowledge gained on different perspectives and assumptions would increase their awareness of the existing and contradicting views when planning or adapting program activities. However, most of the analysis of actors' assumptions, mental models, and potential conflicts of interest did not occur during the actual meetings with the invited stakeholders, but rather in discussions after these meetings. Deeper analyses of stakeholders' mental models and the potential effect of contradicting assumptions did not occur as often as the discussions aimed to reveal or clarify them. We found no indication that this principle was used with the higher-level regional decision-makers, whose assumptions can affect the program implementation. The three main action strategies identified are described in Table 5.

**Table 5:** Principle 3—surface assumptions expressed in practice in the WHCP program.

**Principle**	**Action strategies**	**Detailed description**
P3 surface assumptions	Clarify the underlying assumptions of the policy agreement and about the WHCP program	The strategic plan (Figure 1) developed by the program team was based on an interpretation of the text in the basic agreement and the additional agreements. The input was asked for in meetings with regional representatives, contact persons, professional organizations, and key actors from the National Quality Registries. This was a process of uncovering the political assumptions behind the program and surfacing and integrating the operational and professional perspectives. The range of perspectives represented by the program team members aided the process. The plan became an important tool for communication and for uncovering assumptions about the program held by various actors.
		Clarify the role expectations of the regional contact persons	Partly due to turnover among the contact persons, a need emerged to identify their assumptions, especially the new contact persons, to quickly get them into gear. This led to discussions on the expectations of the contact person and the regional conditions for fulfilling this border role. An introduction kit for new contact persons was developed to aid their enactment of the role.
		Invite stakeholders to discuss issues, challenges, and strengths of the program	The program team invited stakeholders (e.g., representatives from national authorities or staff working with related national agreements) to discuss issues related to the program with the team and with the regional contact persons, and thereby provide their views and perspectives.

### 3.4. Principle 4—reflect and learn

The principle Reflect and Learn concerns continually reviewing new information, assumptions, and learnings to jointly be able to develop and refine a shared vision for, in this case, the improvement of women's sexual and reproductive healthcare. An important part of this principle is to create environments where people are encouraged to reflect and learn.

Initially, the program team focused mostly on spreading information about the program and less on creating opportunities for mutual interaction. However, the focus shifted and efforts to create opportunities and arenas for reflection and learning increased over time. We found many indications of the use of this principle in the program activities and in the described action strategies (see Table 6). The arenas and opportunities to review new information, reveal assumptions, and reflect and highlight lessons learned increased over the program period. From having contact persons meeting twice a year to meetings every month plus two 2-day conferences each year. This development within the program team resulted in the discussion of if and how suggestions, activities, and solutions stemming from different actors, and their perspectives should be incorporated into the new yearly agreements or in the implementation of the program. The arenas and opportunities used for reflection and learning were perceived by program team members to have increased the capacity for change among actors in the regions. Mutual reflection and learning were adapted to the medium used for the meetings (face-to-face or digital meetings) and the time restrictions (length and regularity of meetings). The known effects of the team's attempts to enhance learning to aid program implementation in the regions are limited. The five main action strategies identified are described in Table 6.

**Table 6:** Principle 4—reflect and learn expressed in practice in the WHCP program.

**Principle**	**Action strategies**	**Detailed description**
P4 reflect and learn	Enhance open reflection in groups	Open reflection in smaller groups at the beginning of each meeting with the contact persons. During these sessions, the contact persons can share thoughts and discuss current issues in their regions and their need for support. The small group discussions are followed up in the large group, and suggestions on actions are discussed. This approach became more important during the pandemic.
		Provide national and regional arenas and opportunities for reflection and learning with the regions and their actors	The dialogue meeting tours, where program team members visited the regions to discuss the program intentions, results from National Quality Registries and patient surveys in relation to the current regional situation, were planned to provide opportunities for discussions, reflection, and mutual learning. This same approach was used in the contact persons meetings including open reflections in smaller groups. National conferences with interactive workshops for a broader target group were organized regularly. These workshops focused on, e.g., how to develop care together with pregnant women and their families, create equal care, use staff competence wisely, or develop an integrated care process. The mix of participants created opportunities for people with different perspectives to meet and reflect together.
		Offer courses and seminars	As part of the program, there were offers for regional staff to, without any costs, participate in courses and seminars covering subjects relevant to change and development (e.g., leading change, service innovation, and analyzing and using data for improvement from the National Pregnancy Survey).
		Encourage and enhance reflection and learning within the program team	From the start, the team allocated time and resources for sessions dedicated to team learning and team building, e.g., one Inspiration Day per semester each year. In addition to weekly operative meetings, the team had monthly half-day meetings, which created opportunities for in-depth discussions, reflections, and mutual learning about subjects that could be suggested by any team member.
		Invite external researchers to follow the program process, provide feedback on findings, and summarize learnings	Researchers were invited to follow the program using a learning and action-oriented approach. The researchers provided feedback to the team and to regional partners during the program and identified and summarized learnings to be used in future national policy programs and research publications.

### 3.5. Principle 5—find leverage

The principle Finding Leverage concerns identifying solutions, innovations, and actions that are efficient, have a high impact, are sustainable, and meet the needs of the community, in this case, those delivering care to women in all regions before, during, and after childbirth. The solutions should be based on data, address “upstream” factors, be tailored to the local context, provide support, aid coordination among involved partners, and improve infrastructure (in the area focused on).

Finding high-leverage solutions to the many issues in the WHCP program that could have a high effect nationwide, and in the complex settings of 21 different regional systems, was important but challenging for the program team. Efforts were made to identify and analyze problem areas, spread existing knowledge, and successfully test innovations in each of the program's main goals and strategies (Table 7). Analyses of issues and identification of gaps and knowledge were done both to seek understanding (P2) and to find successful solutions (P5). The generated reports formed a basis for other activities, e.g., webinars and workshops. Developing the regional capacity to facilitate innovation and improvement and to spread and sustain high-impact solutions was identified as important to enable regional actors to implement the changes needed in the program's focus areas. However, although the interrelations among the focus areas and how the issues could be tackled in integrated ways were sometimes discussed within the program team and briefly together with other actors, potential solutions or examples of them were not made as explicit or clearly presented in meetings as the solutions found on single issues or within single focus areas. The five main action strategies identified are described in Table 7.

**Table 7:** Principle 5—find leverage expressed in practice in the WHCP program.

**Principle**	**Action strategies**	**Detailed description**
P5 find leverage	Identify issues, gaps, and areas in need of improvement, and knowledge on the best ways to improve and summarize them in reports.	Reports on issues, gaps, and potential solutions were based on data from the regional reports, interviews, quality registries, and the National Pregnancy Survey, e.g., mapping the clinical pathway before, during, and after pregnancy, current patient characteristics, and improvement needs in the 21 regions, and identify good examples potential successful approaches for decision-makers to manage these needs.
		Spread knowledge, methods, and good practical examples of how to deal with issues and gaps via meetings, conferences, and webinars	The main arenas for spread were contact-person meetings, national conferences for regional actors, and a webinar series. Efforts were made to spread existing evidence-based knowledge on specific clinical methods expected to have a high impact once implemented. For example, a method to prevent perineal trauma during labor found to be successful in other countries was presented at a national conference in 2018 that spread to many regions. Reports were also used as a basis for a webinar series covering several multi-faceted issues and targeting staff in maternity care, antenatal, and post-partum care. The aim was to spread knowledge and examples of good practice solutions to challenges identified by the team or the contact persons.
		Highlight upstream improvement areas that affect other program areas—e.g., the shortage of midwives	A pervasive problem concerned with attracting, recruiting, and retaining competent staff. There is a shortage of midwives in all the regions in Sweden and managing the turnover of competent healthcare staff is an increasing challenge due to demographic changes. This issue affects the work of improving several program areas. During the program, this issue was regularly addressed in meetings with regional actors, and combined solutions were discussed.
		Increase the regional capacity and knowledge about how to facilitate innovation, improvement, and change	Developing the regional capacity to facilitate innovation and improvement, and to spread and sustain high-impact solutions was identified as important to enable regional actors to implement the changes needed. Examples of activities in this area were the offer of a free 3-day course in leading change, workshops, and a course on how to work with service innovations, and seminars and discussions on ways to achieve learning and change with input from researchers.
		Use the strategic plan as a guide for overarching improvement strategies and goals	The strategic plan presented an overview of the program's focus areas and main improvement strategies. The team used the plan as a guiding tool to find, organize, and spread existing knowledge and, to some extent, identify potential high-leverage solutions to issues within each program area.

### 3.6. Principle 6—manage resources

The principle Manage Resources concerns levering and coordinating resources in terms of funding, people, technology, and equipment. To manage resources means to allocate them in a strategic way, so they support any chosen intervention's impact and follow-up on the results. This can, for example, involve temporal aspects, choices of high-leverage solutions, or prioritizing between target areas.

In the WHCP program, the needed changes outlined in the national policy agreement were to take place in, and ultimately be managed by, the self-governing regions. This limited the mandate of the program team to manage resources in relation to the change process, compared to what might be the case in organizations. Since the program was based on a series of separate, but related, policy agreements between the government and SALAR as a representative for all the regions, funding varied over time as new agreements were settled. The program team at SALAR received funding for coordinating national activities to support the regions' improvement efforts, but the main part of the resources was directly transferred to the regions, based on their population size. The regions then allocated these program resources according to local needs and regional priorities. Thus, in practice, the national level had little control over how the resources were allocated and had to find ways to get information from the regions. Initially, the program's reporting requirements were neither very strict nor detailed, but successively requirements changed and increased, and the regional activity reports gained more importance over time.

The national level had two primary means to influence the allocation of resources within the regions. The first was the selection of indicators from National Quality Registries and the National Pregnancy Survey used for follow-ups and presented to the regions. The other was the design of the questionnaire-like template for the regions' yearly activity report, highlighting the importance of thinking about the whole change process, including how the resources were used. Therefore, the program team also offered support sessions to the contact persons when it was time to compile the activity reports. For the 2021 agreement, there was a specific request for detailed information on how the funding had been used in the areas highlighted in this agreement. The four main action strategies identified are described in Table 8.

**Table 8:** Principle 6—manage resources expressed in practice in the WHCP program.

**Principle**	**Action strategies**	**Detailed description**
P6 manage resources	Develop a mixture of methods for following the regional work and program progress	The program team's awareness of the restrictions in terms of managing resources led to a focus on follow-ups of the regional work and progress and the gradual development of a mixed way to select and use collected information to influence resource allocations and direct them to important regional improvement areas, via content in meetings, dialogue tours and summaries of regional activities.
		Gather and analyze yearly reporting from the regions on how they have used the program resources	The regions reported yearly on how the resources were used, provided information on interventions, and reported on their effects. These activity reports provided timeframes and detailed qualitative information on all activities (completed and ongoing) and estimations on how much funding from the program had been used for activities that had ended. The program team compiled the information provided by the regions in yearly reports. For the 2021 agreement, there was a specific request for detailed information on how the funding had been used in the areas highlighted in this agreement.
		Use the region's yearly reporting to help the region get an overview of funding and activities	The reason for increasing the level of detail in the follow-ups as the program progressed was not primarily to influence how the regions distributed or used the resources locally. Instead, the main purpose was described as a way to collect and compile information to be able to help the regions see their own regional investments in a larger context and how these contributed to the development of maternity care from a national perspective. Another aim of the framing of the questions in the yearly report template sent to the regions was to aid and motivate key regional actors to work with improvements in a systematic way.
		Summarize and send yearly program reports to the Ministry of Health and Social Affairs	The Ministry of Health and Social Affairs as one part of the agreement followed up on the activities initiated by the program team each year, which also included the researchers' yearly report.

### 3.7. Principle 7—respond rapidly

The principle Respond Rapidly concerns taking actions and continuously improving solutions or discontinuing unsuccessful strategies, catalyzing action among stakeholders and partners, and re-evaluating when needed. A long-term, nationwide program in a decentralized setting that involves many regions, organizations, and people puts special demands on the interaction between the national and the regional and local levels.

The close interaction that the program team developed with the regional representatives led to expectations on the team to respond swiftly to highlighted problems and needs, expressed in interviews and during meetings. The strategies, both for how to find signals and how to respond to them, constantly evolved as information was accumulated and needs discovered, based on discussions with involved actors, mappings, and data from the National Pregnancy Survey and the quality registries. The three main action strategies identified are described in Table 9.

**Table 9:** Principle 7—respond rapidly expressed in practice in the WHCP program.

**Principle**	**Action strategies**	**Detailed description**
P7 respond rapidly	Pick up signals and respond to feedback from various stakeholders	One example of the ability to pick up and use information from various types of stakeholders was the development of one of the National Pregnancy Survey. The process involved collaboration with a wide variety of actors on national and regional levels. The survey went through several changes and adaptations, both before and after it was tested and launched. Changes were made to adapt the survey, information to end-users, and the IT infrastructure, to the needs and comments of partners and stakeholders, often based on input from healthcare professionals and end-users. Another example is during the dialogue tours where the team asked for feedback on the national support and activities—and could provide rapid responses to clarify, adapt, or change planned activities or pick up new ideas.
		Ask for, respond to, and act on feedback from regional partners	One example of adaptation due to feedback from regional partners is the successive changes made to the template for the regions' yearly activity report, which was adjusted several times during the program to become more user-friendly, to fit with new policy agreements, and to provide the information needed for evaluations.
		Use agile consulting approaches to quickly identify contextual changes affecting the program	The COVID-19 pandemic 2020–2022 was one major contextual factor influencing maternity and delivery care. To adjust program activities to the unfolding situation, a strategy to frequently consult with the contact persons on the current situation in their regions was used. Most of the planned real-life meetings were then transferred to digital format, and new meeting formats evolved. The strategy was also used to discuss aspects that could contribute to, or affect, the status of issues connected to program areas, e.g., post-partum care or a midwife's work situation. This regular interaction enabled the program team to pick up signals and respond quickly to changes affecting the program.
		Respond quickly to contextual change affecting healthcare	A quick adaptation of planned program activities happened in 2020–2021 due to the COVID-19 pandemic. The pandemic had a major impact on the healthcare system and on maternity care, e.g., during periods of high infection rates in the regions the partner could not accompany the pregnant mother to visits, nor participate during the delivery.

**Table 10:** Principle 8—translate findings expressed in practice in the WHCP program.

**Principle**	**Action strategies**	**Detailed description**
P8 translate findings	Use a customized compilation of regional findings in the regional dialogue meetings	In the dialogue tours from 2018 onward, the program team visited each region to meet with decision-makers and contact persons to discuss the local implementation and issues related to the program, the regional findings from the National Pregnancy registry, the quality registries, and the regional activity reports. The team produced an overview in a few pages where a selection of basic information, good and poorer results was presented and visualized.
		Synthesize information in the region's yearly activity reports	The public activity reports were since 2018 synthesized and shared with partners, stakeholders, and the public. From 2020, the reports were structured according to the categories in the strategic plan. A work group within the team synthesized and packaged information on what was going on in each of the strategic areas and compiled summaries of each region's activities. This summary report evolved, partly as a response to the discussions with contact persons on the usefulness of reporting. The summary report was shared with all regions and openly available on the SALAR website, as all reports were produced within the program. Reports were presented and discussed in meetings with contact persons and used to judge which improvement areas to focus on the following year.
		Summarize findings, e.g., from the yearly regional activity reports—and present them in webinars	The open webinar series used findings from National quality Registries, the National Pregnancy Survey, and the regions' activity reports—to illustrate improvement areas and important themes, and present categories and descriptions of good examples.
		Engage a communication officer in the program and establish a national network of regional communication officers	The communications officer in the program team played a vital role in supporting both the team and the regional actors. This person aided the team when producing reports, websites, and films and in the process of producing information to women about the National Pregnancy Survey and templates for regional reporting of the results to various target groups. A national network of regional communication officers was established to strengthen the ability to translate findings and disseminate and package relevant information to regional target groups.

### 3.8. Principle 8—translate findings

The principle Translate Findings means synthesizing and sharing relevant findings, data, and information with partners, stakeholders, and the public. In this process, key partners and stakeholders will be engaged to determine the importance of information and how to spread it. This process can be more or less of a challenge, depending on the complexity of the problems addressed, interventions used, data collected, and settings where information and findings shall be disseminated. In a large improvement initiative such as the WHCP program, this was a rather complex task.

The intention to translate relevant findings, i.e., prioritizing what information was important to share, with whom, how, and why, together with key actors, permeated the entire program. The ability to translate and use findings in the program increased over time, as more data became available via National Quality Registries, the National Pregnancy Survey, and the yearly regional activity reports. Team members described the use of findings and data to support both improvements and learning. Sharing of data and findings created both an interest in and a better understanding of the program and its focus, challenges, and effects on both partners and stakeholders and in media.

## 4. Discussion

Aiming to increase the knowledge of how ST is used in practice in national policy programs addressing wicked problems, we searched for indications of ST in data describing the main program activities and action strategies in a national program addressing complex issues in women's healthcare in Sweden. We used a conceptual framework comprising principles of organizational level ST (22) as an analytical tool, and we have provided narrative examples and descriptions of action strategies used in the program for each principle. This differs from the study of Wilkins et al. (22), which focused on organizations working with the implementation of policies, and their work on identifying and testing quantitative indicators of the operationalization of the ST principles in these organizations and within the area of injury and violence prevention. In this study, we have tested a way to retrospectively identify whether and how ST principles were used (intentionally or unintentionally) within a national soft-law policy program where ST had not been discussed or intentionally introduced as a strategy.

The proposed ST principles (22) may seem logical to follow for any project manager. However, the complexity and dynamics of the policy program (its content and organization), the decentralized healthcare system setting, and the multi-dimensionality of the problems addressed pose additional challenges to actors involved in the implementation of the studied program. Thus, the application of ST on a system level is more complicated than in most (single) organizational settings. Initial understanding and analyses of the system, the issues addressed, and the program features are a foundation for being able to identify stakeholders and important actors to initially involve before considering the other ST principles.

### 4.1. The use of systems thinking in practice within a national policy program

Improving healthcare, or the health of the population, means dealing with complex issues or wicked problems (2, 3). It is difficult to create a holistic view of a complex program aiming to improve several ill-structured issues and induce changes in a large healthcare system. Similarly, it is difficult to design activities for supporting such changes, since it requires considering multiple perspectives, stakeholders, subsystems, and transformation and adaption processes. The ST principles provide some main categories that can aid the classification and description of the action strategies used in the WHCP program. Some main learnings may aid future attempts to understand and facilitate the use of ST in practice when implementing complex policy programs addressing ill-structured and wicked problems in large and decentralized healthcare systems.

#### 4.1.1. The use of collaborative approaches and ways to shared mental models

To convene actors with a different perspective (ST Principle 1) involve them as partners or use collaborative approaches are not exclusively connected to ST or a policy program. Collaborative approaches are the most often promoted ways to tackle complex issues and ensure that important perspectives of those affected by changes and those that can affect them are incorporated into interventions (56). Individuals and teams involved in the core of developing and implementing national policy programs will have to make decisions, solve problems, and use sense-making to create momentum in change processes, but due to the inherent complexity when addressing ill-structured and wicked problems in a complex system, achieving change is a collaborative challenge involving more actors than in organizational change attempts. To comply with national soft-law policies is not mandatory, but research has shown that the Swedish regions find it hard not to participate in national policy agreements (9). Reasons for this can be compliance mechanisms, such as peer pressure and a sense of moral responsibility, and also SALAR's role as an intermediate actor, i.e., being both the region's representative on the national level and a contracting party in the agreement (9). In this case, there was a shared awareness of the problems in women's healthcare and a readiness for improvements among the regions. The regions also had a high degree of freedom to choose which interventions to focus on within the policy program, based on their own needs, and there were no strict performance requirements or target levels as in some of the previous national agreements [e.g., (51)].

The mix of actors involved aided the processes of understanding and identifying leverage that integrated the perspectives of multiple levels. Engaging actors with decision-making power in the ST processes was also a way for the program team to indirectly try to influence the allocation of resources to enable the intended changes. Still, it was difficult to reach higher-level decision-makers, and the use of separate regional dialogue meetings with a group of regional representatives for each of the 21 regions was one activity that was described as having some impact. For stakeholders, especially higher-level decision-makers, to engage, there needs to be a will and an understanding of the needs and benefits of getting involved in an interactive process of building a shared mental model of the system and the issues to be solved. In a complex program context, it might also be beneficial to further define expectations on a program partner or stakeholder, as their interest and agendas can vary (57). Carefully analyzing and clarifying what types of actors are important to involve and how to involve them can aid the work of a program team. However, the team may need to prioritize and channel their interaction efforts to make the largest impact on the program, especially if resources are scarce.

The composition of individuals in a team leading a program is of special importance. If ST is a guiding approach in large and complex programs, this requires some attention in the initial forming of a team. A clear strategy in the studied program was to include members with different competencies and perspectives, some with connections to other related national agreements, and some with their basic employment in the regions. It is unlikely even for skilled program managers to possess all the capabilities needed to manage a national program focusing on large system transformation. This strategy was also seen as an effective way to extend the team's network, improve communication with stakeholders and partners, and promote an understanding of the program as part of a larger system transformation, i.e., to enhance ST in the team. Previous research on program management has focused on individual program managers and their competencies and actions, but less is known about the nature of the distributed capabilities among other actors in the core and extended program team and how they may contribute to a more holistic view of the program and its change process (58).

The need to address complex and interrelated issues and wicked problems in healthcare in Sweden or elsewhere is not new, but there has been an increase in more complex national initiatives over time. The WHCP program is one example where the goals concern development in a diversity of areas, comprising great challenges. Challenges faced by decision-makers, care providers, and patients may be similar in a general sense, but the dynamic regional and local conditions must be considered when aiming for more sustainable changes (59). Thus, the program team had to consider assumptions and mental models held by actors on multiple levels and develop strategies to connect these views. This was difficult, but the strategic plan, represented also in graphical format, played an important part in this process in several ways. First, by involving stakeholders in the development of the plan, i.e., operationalizing the political intentions expressed in the agreement, which helped to develop a shared vision of the program and its goals, and second, by functioning as a visual communication tool for the team (both internally and externally). Using visualization to represent concepts, components, and their interrelationships is a powerful methodology within ST that can aid sense-making and the creation of shared mental models (45). Even so, to reveal underlying assumptions in general, and especially of important strategic-level stakeholders and decision-makers on higher regional levels, was less described as a strategy by the program team. Also, higher-level regional managers were hard to reach to inform about and discuss the program, and revealing assumptions would require more interaction. Instead, the program team focused on other regional actors easier to access, such as the contact persons.

#### 4.1.2. An iterative learning strategy and ways to aid the development of multi-level interventions

In a large, complex, and long-term policy program, there are many dimensions and conditions that must be considered to enable reflection, learning, and collaboration among the involved actors. The challenge is to create opportunities and communication arenas that can support collaboration, reflection, and learning, and find and develop ways to deal with ill-structured and complex issues. Achieving deeper learning and changing people's behavior and action strategies takes time. This requirement may not fit very well with the restricted time and/or resources of a program, or with the expectations and views of involved stakeholders and partners.

An important aspect of the program was to provide opportunities and arenas for reflection, feedback, and learning, on group and individual levels. The frequent use of group discussions in meetings is one example. In meetings, there was often a mix of participants from different levels of the healthcare system, which enabled learning and exchange of experiences across national, regional, and local levels. Sometimes, single participants have multiple perspectives, e.g., a regional contact person could also be involved in national groupings, such as producing guidelines. Altogether, the large number of activities designed for enabling interaction, reflection, and learning, such as network meetings, the teams' regular half-day meetings, and numerous workshops and courses, can be interpreted as representing a learning culture within the program, especially on behalf of the program team and the contact persons' network. Active reflection and learning opportunities are at the core of a change process, especially when aiming to achieve double-loop learning for more substantial behavioral changes in both individuals and organizations (60, 61).

Synthesizing and sharing relevant findings and using them to enhance learning and change was a core task for the program team. Interactions and relations with partners and stakeholders were central to the program's communication strategy, which emphasized responsiveness and an adaptive approach regarding how to reach different target groups and audiences. Strategic communication within the program involved a meta-process of integrating information, understandings, and learnings on the program level, making sense of the results in a larger perspective, and choosing the best way to package the information and feed it back to key actors. Management of such processes requires ST skills (30, 33).

One aspect of the learning approach applied in the program was to engage a variety of actors in developing interventions that could affect and improve issues identified in each program focus area. ST has been suggested to aid the process of identifying high-leverage solutions that can address multiple health outcomes (11). The program focused on many interconnected challenges. Finding leverage and multi-level solutions that can affect the whole system and its sub-parts is seen as important (14), but in the decentralized Swedish healthcare context with 21 autonomous regions, it presents a real challenge. The program team used a strategy with iterative reflection and learning loops to build joint understandings and consensus on problems and collaborative approaches to search for interventions to improve issues that could be adapted to various regional and local contexts.

### 4.2. Multiple and interrelated levels and dimensions of ST in complex policy programs

Understanding ST in use in a policy program involves an understanding of the overlapping nature of the hierarchical system levels in the program context, i.e., national, regional, and local system levels (Figure 2), and the interactions among the organizational, group, and individual-level ST potentially at play in the program strategies.

In the WHCP program, it was important to achieve a holistic view and a common understanding of the program issues among actors in different parts of the system, e.g., politicians, authorities, and professional organizations on the national level, and politicians, public management, and care providers in the autonomous regions. Even so, in-depth discussions to identify gaps between mental models together with partners were less frequent and gaps would typically become evident after some time and discussed in other group constellations. Reasons for this might be the complex political landscape and the dual role of SALAR as both the coordinator of change initiatives stemming from the government and the organization representing the rather independent regions and municipalities (9, 53).

**Figure 3:**
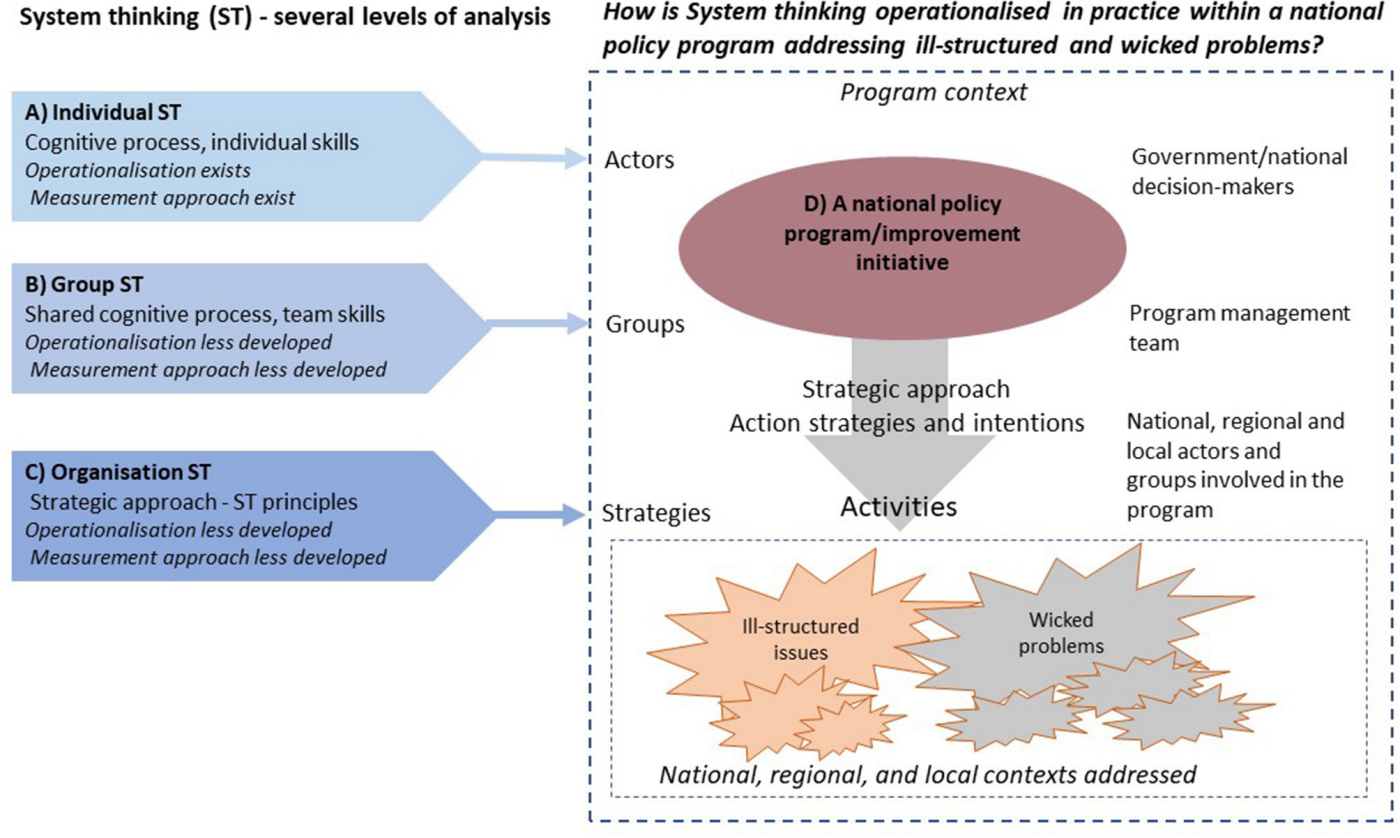
Systems thinking (ST) on several levels of analysis in relation to a national policy program.

Much of the previous research on how ST can be used in practice has focused either on the individual, group, or organizational level. Combined approaches are scarcer but exist [e.g., (43)]. Figure 3 shows the system levels involved in a large healthcare policy program and examples of aspects influencing ST on each level. The community/society level can be added, but it remains to be seen if ST can be investigated on this level. However, the wider national context and its structure and culture will have an impact on policy programs, and the external context of the healthcare system addressed in such a program must be understood. Among other things, ST highlights “the importance of coordinated and effective interventions across multiple levels of change (e.g., individual, organizational, community) (…) and the critical role of strategic communications to catalyze, coordinate and support change” [25, p. 154–55]. Our studied case provides some practical examples of these aspects, in terms of the action strategies used by a policy program team.

One reason for the limited empirical studies of the use of ST in practice, especially within public health (15–17), might be the complexity of a combined approach and the difficulties of comprehensively presenting such studies. Looking at the implementation of a national policy program from an overarching system perspective, it is evident that ST may be used at the individual, group, and organizational levels simultaneously (Figure 3), and that an integrated approach is needed to understand how ST is expressed in practice, how its use can be supported, and assessing the possible impacts of using ST to facilitate change on different levels (individual, group, and organizational levels). For example, it seems important to actively choose a person who possesses ST skills as a program manager, to foster collaborative ST in program teams and regional teams, and to develop action strategies in line with organizational-level ST.

### 4.3. The usefulness of the framework's principles for identifying ST in practice in complex policy programs

A general observation when applying the ST principles to our qualitative data is that the principles are somewhat overlapping. A holistic view is more evident in some of the principles, and it emerges as the principles are added to one another. Another observation was that as we analyzed the data, we found that multiple principles were enacted simultaneously in each of the main program activities. This study describes the nuances of how ST is used in practice within a policy program context.

It is difficult to judge the effects of the use of the ST principles on the outcomes of the ongoing program as this would require a more extensive understanding of both the issues addressed and the mechanisms underlying the ST principles and the action strategies related to them. In addition, the way ST is used, or not, in the 21 regions needs to be addressed. Also, wicked problems cannot be seen as having linear cause–symptom–effect relationships, they evolve unpredictably over time and involve value conflicts among actors (62). This makes it difficult to assess the impact of ST principles on the WHCP program outcomes; possibly, the impact on involved stakeholders and partners, and their action strategies, could have been assessed, based on additional interviews.

### 4.4. Study limitations

The study is limited to one case, a policy program. The WCHP program was chosen for several reasons: It represents a complex system (14) as it addresses complex issues and challenges in a decentralized healthcare system; it is a longitudinal program where the opportunities to develop ST have been good, and indications on a comprehensive approach have been described in previous reports (in Swedish). In addition, an extensive case study database exists, where indications of applications of ST can be found. However, we did not analyze all the data in the database in this study, as it was not feasible due to its scope and the time available. All interviews conducted with the program team over time were analyzed, but regarding the other types of data sources (e.g., observations and archival data), a representative sample was selected and analyzed. An analysis of the total dataset may have yielded a slightly different or more complete and richer picture of ST in practice within the program. However, the researcher's familiarity with the data and the knowledge gained by studying the program for 6 years guided the selection of data. Theoretical generalization (63) can broaden the use of the study but still, the specific conditions of this example from the Swedish healthcare system must be considered.

## 5. Conclusion

There are some main learnings and implications from using the organizational-level ST framework to identify and describe how ST is applied in practice in the context of a national policy program addressing several wicked problems in a decentralized national healthcare system. Some practical implications may also aid future attempts both to understand and to facilitate the use of ST in policy programs.

First, engaging the right partners in the change process, who represent a broad range of different perspectives and have a mandate to act, is key for enabling ST on the organizational level, but even more so in a national program aiming for impact in 21 self-governing regional systems. Thus, this first ST principle forms the basis for applying the other seven principles described in Wilkins et al.'s framework.

Second, the high degree of complexity of the program content and the variety and dynamics of the settings that a national policy program often encounters create conditions that need special attention from the actors involved. A high degree of program dynamic and complexity executed in a complex program setting require a deeper understanding of underlying principles guiding ST, and more time and effort to plan and execute ST-informed action strategies. The strategies must be used, and adjusted, repeatedly during an extended time period. Such programs will need more resources, time, and competence during their implementation than programs with less complexity.

Third, even very basic use of ST tools (e.g., developing a graphical representation of a strategic plan) can function as important levers for ST in practice in a large policy program aiming for system change. Visualization is a practical tool of special importance if the program and setting complexity are high.

Furthermore, the narrative descriptions of the action strategies related to ST principles provided in this study, as well as the described difficulties encountered by the program team when using them, provide details that can aid others who lead and support the implementation of soft-law initiatives and policy programs.

Detailed, systematic descriptions of action strategies used to support changes in large systems initiatives are still scarce. A multi-level approach is needed to fully grasp how ST is expressed in practice, as individual, group, and organizational-level ST are all inherent in a policy program. To increase the understanding of how to identify and learn more about the practical use of ST in policy programs and public health, we suggest further studies of how ST is used in practice in other policy programs, both in similar and different national contexts. The framework of the organizational ST principles used in this study, together with our observations of the interrelationships between different levels and dimensions of ST in practice, can contribute to such studies.

## Data availability statement

The raw data supporting the conclusions of this article will be made available by the authors, without undue reservation.

## Ethics statement

The study was reviewed by the Regional Ethical Review Board in Stockholm, and they found not to need for a formal ethical approval and issued a statement of this (Ref. No. 2018/620-31).

## Author contributions

MN and HS designed the study and drafted the manuscript. MN, ST, and VS collected the data. MN, ST, VS, and HS conducted the analyses. All authors read, contributed to the article, and approved the final manuscript.
