# Supplementary material for: Systems thinking in practice when implementing a national policy program for the improvement of women's healthcare
Source: Front Public Health. 2023 Sep 29;11:957653. doi: 10.3389/fpubh.2023.957653 (PMC10570416; doi:10.3389/fpubh.2023.957653)
Supplement: Supplementary file 1 [file Table_1.DOCX]

# Supplementary files

## Supplementary 1 – Interview guide - Program team members 2018

*1) Background facts*

1. Tell us briefly about your background.
2. What is your function and role within the WHCP program? What are you responsible for?
3. How did you get in touch with the WHCP program and why were you chosen to work in the national team?
4. How long have you been working in the national WHCP program team?
5. If you think back to the time when you first heard about the program (or that you were going to work within the program) – do you remember what your thoughts were then? Describe them.
6. Who initiated the WHCP program and who has the main responsibility for it (e.g., is there a steering group)?

*2) Interventions/activities*

1. What is your perception of the purpose of WHCP? Why is this investment being made?
2. What is the goal of WHCP, what do you want to achieve?
3. What are your current expectations on the program?
4. What are the main challenges and problems that the program could affect or improve?
5. Which target groups have you worked with or towards so far? Has this changed over time?
6. What interventions (activities) have you carried out so far? Overall order of activities?

*3) Knowledge of the regions' work with and organization around the WHCP program*

1. Can you briefly describe what you know about the different regions in terms of how they work with WHCP and how their work has been organized?
2. Can you describe how the regional WHCP work has developed over time? Which regions started early, and which started later?
3. Are there any examples of successful regional initiatives within the program that you know about and can briefly tell us about?

*4) Strategies*

1. Can you describe how you and/or* the team have worked with the program, i.e., the strategy used so far to reach out and fulfill the program mission?
2. Was what you described deliberate strategies from the start or something that has emerged?
3. Have you changed your strategies and/or have the team strategies changed over time?
4. Tell us more about the actual planning and start-up phase. How was the program initiated and started (and what was your role)? Was what you described a deliberate strategy?
5. What strategies have you and/or the team had for information and communication? Towards whom?
6. What strategies have you and/or the team had for feedback to contact persons and regions?
7. Have you adapted your strategies or has the team adapted their strategies to different conditions in the regions? How?
8. What are/have been the important prerequisites for being able to conduct your work with WHCP?
9. What are/have been the biggest obstacles so far in achieving the intentions of WHCP?
10. Who are/have been the biggest facilitators so far in achieving the intentions of WHCP?
11. What lessons have you learned so far from the set-up on your part? Anything you would have done differently if you could do it again? Something you should have done but didn't do?
12. Is there anything else you want to tell us about strategies you have used or intend to use?

*5) Conditions for change and learning - strategies*

1. What do you think is important for being able to change the way of working when it comes to achieving better care before, during and after pregnancy?
2. What do you think is important for being able to improve the way regions and care givers work with improvements of care during and after pregnancy based on risk assessments and registry results?
3. What do you think is important for being able to change in the way of providing care when it comes to achieving better health for women?
4. How do you see your role in relation to the role of the regional contact persons in the work to achieve the intentions of WHCP?
5. How do you perceive *your role* in relation to managers on different levels in the regions and their roles in the work to achieve the intentions of WHCP?
6. How do you perceive the *role of the contact persons* in relation to managers on different levels in the regions and their roles in the work of achieving the intentions of WHCP?

*5) Reactions and results*

1. What reactions and effects have you seen so far among the contact persons?
2. What reactions and results have you seen so far in the regions?
3. What reactions to implementation have you received so far from those responsible for the WHCP-program (e.g., steering group, persons at the Ministry?)?
4. What reactions to implementation have you received so far from regional politicians and managers?
5. Are the effects that you have seen so far expected or unexpected on your part?

*6) Future plans*

1. What are your and/or the teams’ future plans in relation to the regional contact persons and other key actors?
2. What are your and/or the teams’ future plans in relation to the regions?
3. Are there any plans that link this to other national programs?
4. What factors do you consider to be decisive for the success of the initiative as a whole?

*7) Is there anything else of importance that you want to convey? Something we haven't touched on?*

*team members were here given the opportunity to present both their own strategies and experiences and the perceived team strategies

## Supplementary 2 – Interview guide - Program team members 2020

*1) Background facts*

1. What is your function and role within the WHCP program? What are you responsible for?
2. How long have you been working in the national program team?

*2) Strategies*

1. Can you outline how you have structured the work at a national level and the strategies that you have used (i.e., as a program team) to support the implementation of the WHCP so far*? (follow-up on details if needed for clarification*)
2. What are the main *conditions* that you (i.e., as a program team) have considered or adapted to so far?
3. What have been the main *facilitators* so far for the work at national/program level (i.e., for the program team)?
4. What have been the main *obstacles or difficulties* so far for your work at national/program level (i.e., for the program team)?
5. How is the Strategic Plan used by the program team at national level?
6. More details about the content of the Strategic plan.......how do you (as a team) work at national level with the goal areas x-x and the strategies x-x described in the plan?
7. Do you know if or to what extent the Strategic plan is used within the regions? Can you describe some examples of how they use it?
8. Did you (i.e. as a program team) try to connect with and get support from other national programs?
9. If yes - Which ones?
10. If yes, what did you do?
11. If yes - How is it going/will it have effects on the programs or the regions?
12. Is there learning between different national programs and how is this learning expressed?
13. Are there any plans that link the WHCP program work to other or new programs (other than those mentioned)?

*C) Communication*

1. What overall strategies have you (i.e. as a program team) had for information and communication within the program (i.e. exclusive of the internal communication within the program team)? Against whom?
2. Which communication arenas do you think have had the greatest impact within the initiative? Why?
3. The program team have made physical and digital visits to the regions so called dialogue meetings – how do you think they have contributed to supporting the implementation of the initiative's intentions?

*D) Monitoring, evaluation and learning*

1. What are the most important sources of information for the program team so you know how the work with the WHCP is going in the regions?
2. How does *SALAR's annual follow-up on regional activities* contribute to the development of the initiative? How is it fed back and to whom?
3. How does the information provided by the National quality registers (e.g., the Pregnancy Registry) contribute to the development of the initiative? What do different indicators say about how things are going?
4. How do you think the *Pregnancy Survey* will contribute to the initiative?
5. How does the program evaluations performed by the Swedish Agency for Health and Care Services Analysis contribute to the initiative? How have they participated? How have the reports been used at a national level/by the program team?
6. What role do you think the invited *researchers work* has in the initiative? How has the feedback from the researchers been used so far?
7. Are there any other ways to follow up on what has had an impact in the program that we have not mentioned? (How do they contribute?)
8. What lessons have you learned so far from the set-up on your part? Anything you would have done differently if you could do it again? Something you should have done but didn't do?

*D) Future plans and important factors*

1. What are the plans for the Autumn and next year? Activities planned?
2. What are future plans related to the regions, the contact persons, and other regional key actors?
3. What factors do you consider to be decisive for the success of the program as a whole?

*E) Other*

1. How has the Covid 19 pandemic affected the efforts and work with the program at the national level?
2. Is there anything else of importance that you want to convey? Something we haven't touched on?

## Supplementary 3 – Interview guide - Regional contact persons 2018; 2020-21

*1) Background facts*

1. Tell us briefly about your background and where you are positioned within the organization/region?
2. What is your function and role within the WHCP program in the region? What are you responsible for?
3. How did you get in touch with the initiative and why have you been selected as a contact person?
4. How long have you been working on the WHCP program in the region?
5. If you think back to the time when you first heard about the WHCP (or that you were going to work within the program) – do you remember what your thoughts were then?

*2) Regional organization (overall perspective/level)*

1. Can you describe how the overall work within the region with the WHCP program has been organized? Are there more people than you who work with the program?
2. Has this changed over time? How?
3. How have you worked to spread information about the program within the region?
4. What has influenced how you have structured your work and how the region has chosen to structure the overall work within the region over time? What has been considered?
5. How have you or how do you work with other national programs/initiatives/agreements?
6. What are the future plans for the regional work with the WHCP program?
7. Do you have suggestions on how to optimally organize work with the program within the region?

*3) Prerequisites, facilitators, and obstacles*

1. What do you think are the most important conditions to be able to work optimally with the investment in the region? Do they exist?
2. What has facilitated your work with the program within the region so far?
3. What has hampered your work with the program in the region so far?

*4) Implementation strategy — detailed*

1. Have you worked to link the work within the program to existing goals and strategies in the region – If yes - How?
2. How have you gone about choosing what issues to work with within the program?
3. Are there regional action plans or similar for the program’s focus areas?
4. How do you evaluate the results of selected initiatives and interventions regionally? Is it an accepted practice within the region?
5. Are there people with responsibility for leading and/or following up various regional initiatives related to the WHCP program?
6. How do you report/disseminate information within the region on what is happening in relation to the program and the results of different regional initiatives? To whom and when?
7. Is there any development support or any type of support function involved in the work with the program? If yes - Which one and how is it used?
8. How have you worked to maintain and disseminate good solutions/working methods within the region? Outside of the region?
9. How do you think it has worked so far for your region in terms of the work with the initiative?

*5) National support*

1. How do you perceive the support from the national program level to the regions and the contact persons so far?
2. What has been particularly good in terms of the national program support and activities that you have taken part in?
3. What has worked less well in terms of national program support and activities to support the regional level?
4. How has the national follow-up of the regional work functioned? (*reminder – development from a few questions 2016, a questionnaire 2017 and the developed Excel template in 2018*)
5. What kind of support for regional work with the WHCP program would you like or wish to have from the national program level?

*6) Other*

1. Do you have anything else you want to add that has not been mentioned?

## Supplementary 4 – Observation template and one example

**Meeting with regional Contact persons - Date**

Observer: XX

Participants: Program team: (names)

Participants: Regions: (names of the regions)

| Date: 21 September 2021 kl. 10.00-15.00  Place: Zoom  Target group: Contact persons in the WHCP program |
| --- |

| **PROGRAM** | |
| --- | --- |
| 10.00-10.30 | **Welcome!**  Check-in: What's going on in your region? |
| 10.30-10.40 | **Report on women's sexual and reproductive health throughout life**  (name), Program team SALAR, (name), Program team SALAR |
| 10.40-11.05 | **For the next two years of the initiative:**  Discussion in small groups: Now that we have been informed that the initiative will be extended until 2023, what possible needs or gaps do you see that your region has? |
| 11.05-11.20 | **Break** |
| 11.20-12.00 | **Gathering in a whole group** What have you discussed in the groups? |
| 12.00-13.00 | **Lunch** |
| 13.00-14.00 | **Using the results of the Pregnancy Questionnaire**  (name), Program team SALAR (name), SALAR's working group  (name), SALAR's working group |
| 14.00-14.15 | **Break** |
| 14.15-14.45 | **Development of the Pregnancy Questionnaire**  (name), Program team SALAR |
| 14.45-15.00 | **Closure and overview of what's going on during the autumn** |

Observations

|  | **Agenda** | **Content** | **Comments** |
| --- | --- | --- | --- |
| 10.00-10.30  10 regions  15 people | **Welcome!**  Check-in: What's going on in your region? | Discussion in small groups  Feedback:  *Group 1:* A few weeks ago, (name region) introduced sampling for CTG (cell sample). How was the communication experienced? Have worked a little differently with reference group, remuneration etc. Success factor to work with professional help from communicators – a success factor and the work with reference groups. The importance of self-sampling kits there is info in picture form. However, need to translate into more languages.  (Name region) – we have introduced the same test.  Cultural doula permanent in *(name region).*  *Group 2:* (name region) *–* how it went with the crisis situation agreement – backlash with worn BM who will have their fourth week of vacation – it's a big frustration. Continued turbulence. New manager 4/10 from Halland. We have had very good teamwork at the maternity ward – with the doctors.  *Group 3*: (name region) We talked about the fact that the program continues, there is a need not to change the framework so much but to work long-term with the investments that are ongoing, do not add more things. Aftercare is where we need to get better and get better at involving patients.  *Group 4*: (name region) relatively stable, several new midwives – challenges to get to do long-term planning, many retirees. Otherwise, dark picture in (name region) *and* (name region)– not seen effect of this seeding. Difficult with attractive scheme models. There have never been so many midwives in Stockholm, but they do not work with maternity care. We have attracted them with money/salary, but it no longer attracts. They don't want to work on call, they want to be off with their family. There are shorter hospital stays and at the same time you must do much more, and it becomes very tiring. More effort is needed. Stressful.  *Program team member:* I had meetings with the organizations of the care professions. Three things: Even more person-centered care, more flexible working time models, (care models?), Now we have had the interactive webinar (Glo och sno), for example, about different schedules, working hours and care models. It was the profession’s organizations input. |  |
| 10.30-10.40 | **Report on women's sexual and reproductive health throughout life**  Two program team members from SALAR | *Program team member:* There is no knowledge support for sexual and reproductive health in Sweden that takes in the life perspective. Now we have done a national survey and the report will be ready in November. Many interviews and literature studies. The report is primarily aimed at decision makers.  Input to SALAR from contact person – there should be patient representatives in SALAR's work.  *Program team member*: in what way should they be involved? | PowerPoint (PP) - add document to data base |
| 10.40-11.05 | **For the next two years of the initiative:** Now that we have been informed that the initiative will be extended until 2023, what possible needs or gaps do you see that your region has? | Discussion in small groups  *Program team member:* What do you need to be able achieve success at work with the program?  Discuss and provide input.  Reunion 11:20 a.m. | PP |
| 11.05-11.20 | **Paus** |  |  |
| 11.20-12.00 | **Gathering in a whole group** What have you discussed in the groups? | Group 1: Funds must be secured. Be able to attract staff. Think long-term and continue what we have started. Personnel investments and knowledge management. The importance of national knowledge support; Long-term – The pregnancy survey. Take advantage of what you can continue to work with over time.  Group 2: Continue to work long-term with the program investments. We have slightly different conditions on (name small region). Continue to work with skills supply and co-care (after childbirth). Having to think differently when it comes to staffing. Digital visits are good examples.  Group 3: The pregnancy survey has defined aftercare as an area to develop further. It is organized in many different ways in Sweden – make an elucidation of how people work and have organized aftercare. SALAR can help us develop factors that can aid. More knowledge is needed – how to rig the organization, so it becomes safe and secure. We also have mental illness.  Group 4: Then working time models and care models. We would need help to introduce different types of experiments to try out with them. Not one model that applies all over Sweden. Need to find more stability, not just train new ones who then move. It is important to raise these issues in the HR and Health Care Directors and Regional Directors' networks.  *Program team member*: The National Board of Health and Welfare is in the process of completing its knowledge support regarding aftercare. Then we can bring it up specifically as a theme at a contact persons meeting. |  |
| 11.40-12.45 | **Lunch** |  |  |
| 13.00-14.00  17 participants  12 regions  Region x joins the meeting | **Using the results of the National Pregnancy Survey**  (name), Program team SALAR (name), SALAR's working group  (name), SALAR's working group | **SALAR's working group facilitates the session with detailed descriptions of use.**  Three surveys with a total of 102 questions  Big data. Rating questions scale 1-5 or 1-10. One option – yes/no; frequency estimates; several options; Free text.  Where can the results be found: National level report SKR; Dashboard; Regional Excel file, order it from the Pregnancy Registry.   - Dynamic, replenished as responses come in. - The free text answers cannot be seen by anyone else than their own organization. - Static in the National level report – got results this summer – for Contact persons. - Healthcare professionals can order extracts- Costs money. - Walks through how to access the Dashboard (via the Swedish Death Registry website). - The number of answers below 5 is not shown for personal privacy reasons. - Answers under 10 responses are not reported.   Would you be helped by us producing a new survey file or is the dashboard enough?  Menti (web program) question – to get answers.  A written manual will be produced as well as a video description. | See PP for details |
| 14.00-14.15 | **Break (13:42-14:00)** |  |  |
| 14.15-14.45  14 people | **Development of the National Pregnancy Survey**  (name), Program team SALAR | Program team member**:** Go through the part regarding ‘my genitals’ which is supposed to be used for research.  Replace parts of the survey; add new questions; New themes?  Discussion in small groups   - Caution with changes so quickly - Full functionality after a certain time - Difficult to answer detailed questions – perhaps belongs more in knowledge management area (regional groups exists) - What do we want to do with the survey? What do we want to follow? What do we want out of it? Maybe it is good that if you do not make any changes so you can follow them over time – and let it be as it is. - Maybe not to remove – it will be difficult to follow over time. - Difficult question to consider. What is there – so it does not become a question that is asked unnecessarily. The survey is comprehensive as it is. How much can you delve deeper to get answers in areas? There may already be free-text answers. For this reason, maybe you should wait with any changes – it feels a little early. We need to get a whole picture and work with the issues a little bit.   *Program team member*: Summarizing – you are hesitant to remove questions, and want to follow over time, as well as doubts about how to ask questions e.g., about aftercare. It is, of course, important that what we are asking for is also something that we can improve. Thanks – a little to digest. We will talk to the various expert groups and will get back to you. |  |
| 14.45-15.00  14 people | **Closure and overview of what's going on during the autumn** | 1/10 seminar for regional politicians and decision-makers.  Glo & sno webbinar: 1) Meeting staffing needs, 2) why do people quit in the spring, 3) aftercare 4) digital ways of working.  During November – gap analysis presented "Women's health from a life cycle perspective.  December extraction of national results postponed until early 2022 |  |
|  | **After-discussion** | After-discussion about how and why you want the pregnancy survey figures (dashboard or Excel). Thinking that it can be different around different target groups – more business-related – dashboard and more strategic/regional overview - excel | *Program team (only) reflects on the discussion during the meeting* |
