# Supplementary material for: Systems thinking in practice when implementing a national policy program for the improvement of women's healthcare
Source: Front Public Health. 2023 Sep 29;11:957653. doi: 10.3389/fpubh.2023.957653 (PMC10570416; doi:10.3389/fpubh.2023.957653)
Supplement: Supplementary file 2 [file Table_2.DOCX]

**Supplementary 5.** Examples of data from the WHCP program in each of the eight ST principles in the Wilkins et al’s framework (23).

| ***1. Convene Partners*** |
| --- |
| **Interviews, program team members**  **2018:** “It is important to cooperate with different national authorities, and we do so regularly. We invite the professional associations to dialogue meetings, e.g., the Swedish Association of Midwives and the Swedish Care Association. Our contact at the NBHW also works with the National Program Area for Gynecological Diseases and Childbirth. We are in contact with e.g., the Swedish Public Health Authority.” |
| **2018**: “We have a structure [for convening partners], we analyse our potential target groups and how we can get invited to them, and if we do not get invited, we invite ourselves, for example to the Healthcare delegation an important forum of [regional] politicians. We try to be active. We have invited persons from NBHW and invited ourselves to the Ministry. You need to think, who is in a strategic position?” |
| **2020:** “The team has people with different competences from different units and thematic areas within SALAR to mirror the importance of working together across areas. We have members from the sections of healthcare, and health and equality which focus on public health and living habits. Others are from the section for employer policy, focusing on issues about staffing (high turn-over, lack of staff) and how to secure adequate competence. There are contact persons appointed by the director of health in each region. The regions need to create good conditions for them so that we can interact with each region through them.” |
| ***2. Seek Understanding*** |
| **Interviews, program team members**  **2020:** “To make gap analyses and point out the gaps for the regions is important. The agreement is designed so the regions themselves can prioritize and ask what gaps they have. For their support we have used surveys and constructed a strategic plan. The plan is a success. It makes it easier for us to see the gaps at the national level. Pushing development is partly about pointing out gaps and stimulating opinions.” |
| **2020:** “During the 2018 dialogue tour, we immediately got a picture where [the regions] were and what they needed. […] It is a good way, in a smaller scale and with time to talk about a particular region and their challenges. In two-hour meetings we focused on program efforts from their and our perspective, and what support we can provide. This year [2020] the dialogue tour is via video meetings [due to the pandemic].” |
| **Archival data 2017, Invitation to a national workshop in October 19, mixed audience** Together for a strengthened supply of skills in maternity care and an improved care chain. We will together discuss issues and identify solutions for the future supply of skills in maternity care. How can the care chain be developed to meet the needs of different patient groups? How can the attractiveness of professions in maternity care increase? What is required of management to achieve a long-term sustainable supply of skills? *Target:* Managers, (care unit, medical care, or operations managers), people working with HR within maternity care. |
| **Archival data 2018, Regional dialogue meetings with a mix of regional representatives**  *Questions to be discussed:* 1) What characterizes a well-functioning care process? 2) What are the challenges in your region (e.g., linked to competence supply, continuity, information, communication)? 3) What measures need to be taken at different levels (regional/local/national)? 4) How can the program team best support you in your continuing development work? |
| ***3. Surface Assumptions*** |
| **Interviews, program team members**  **2020:** “We have been proactive and invited other national authorities to ask what they think before our plans are set, to give them a chance to influence the design of quality improvements initiatives. It has been very appreciated. We have digital meetings with the NBHW, the Swedish Public Health Authority, and the Swedish Agency for Health Technology Assessment and Assessment of Social Services. The strategic plan is an example where the professional organizations, the national quality registries, and the regional contact persons were invited. It is a successful way to work. The process is as important as the results.” |
| **2020:** “We have a program team with different competencies, which is extremely important and enables us to see questions from different perspectives. Our new team member is impressed by the climate and culture in the team. ‘I'm not really used to this, it's amazing’ she said. ‘It is okay to have different views on the issues we work with’.” |
| **2018:** “I can deliver inputs from another perspective. If we [the program team] are proofreading a report, my input is appreciated. My role sometimes is to be the outsider, to think and see things differently” |
| **Archival data 2018, Invitation to a program team meeting** Goal of the day: To create consensus, gain understanding of the big picture of the program and the continued work ahead. Preparations for the meeting: 1) Read through the Strategic plan and the program agreement, 2) Prepare your questions – high or low, everything you wonder about in the program or perceive as unclear. |
| **Observations 2019, Contact person network meeting**  Discussing the strategic plan: “There are often different logics behind work with quality development. For example, person-centred care requires major changes. It is a challenge to stand before the staff and say that we should have a more person-centred care, equality, etc. The strategic plan and its visualization are very useful communication tools for managers in those situations.” |
| ***4. Reflect and Learn*** |
| **Interviews, program team members**  **2020:**"In network meetings there are many people, we don’t know whether everyone dare to speak. Therefore, smaller dialogue meetings are important. […] During the tour we found out that all regions had problems choosing issues to focus on. They wanted us to tell them, but we replied that we couldn’t since regions face different challenges. We then asked ourselves ‘how can we provide general help and find the program cornerstones, even if the regions will choose focus? [based on this we developed the strategic plan]. We have produced several reports and guides in different areas, in a neat, educational way.” |
| **2020:** “The strategic plan is the base for our work. We continuously go back to it and ask ourselves in what way our work contribute to the whole picture? This is great, working on separate parts can give good results, but combining it, as this plan does, enables us to contribute to the whole. Many small things need to be done to move the whole program forward. It is like a fish in a shoal - when everyone works together in the same direction it becomes like one big creature. The strategic plan contributes to this wholeness.” |
| **2018:** "It is right to prioritize face-to-face meeting because they serve an important purpose - to connect ‘ambassadors’, i.e., real people, to a project. This is most rewarding, as it is people who can transfer things to conversation topics in lunchrooms and at meetings and talk about them with colleagues who face the same challenges or share the same interests. I believe in personal meetings with our target groups.” |
| **Archival data 2019 National conference** *Target:* Managers, quality developers, HR-officers and others who work with the care of women from pregnancy to childbirth, including neonatal care, and other designated people in the regions. *Purpose:* To share knowledge and experiences about the on-going work to improve the care of women and their families before, during and after pregnancy throughout the country. The day offers both lectures and interactive themed rooms where participants can discuss and be inspired by the work of others |
| **Archival data 2018, Invitation and agenda for a contact person meeting** “The purpose of the day is to provide the contact persons with the opportunity to meet and exchange experiences on their work with the agreement. Our hope is to create a good climate for learning between regions and SALAR to contribute to improvements in maternity care and other care related to women's health" |
| ***5. Find Leverage*** |
| **Interviews, program team members**  **2020:** Absolutely [learning takes place between national programs]. I am a bridge between ventures, I talk about the program in other contexts. Our report on how to develop ways of working with examples from women's health has been spread in HR networks, a lot is generic so others can benefit. We talk to municipalities about how [the program] is related to their development initiatives. The report connects to work done in [municipality x]. A podcast section based on the report. All is located on our website targeting people in all areas. Municipalities have workshop material that we link to. We connect everything.” |
| **2018:** “There have been many good examples [presented in webinars]. […] Such as when a maternity care unit set up a tablet so that patients can check in themselves when they arrive - that freed one staff for other tasks. These are good changes, even if they may seem small. |
| **Observations 2021 – Program team meeting** *Discussions in webinars on learning from good examples*: "The webinar on digital working methods got good evaluations. There were two practical examples of interactive digital meetings with parents. One of how public caregivers in maternal healthcare can organize digital care meetings, provide initial help to get started and as a group leader in interactive meetings ". |
| ***6. Manage Resources*** |
| **Interviews, program team members**  **2020:**”For sustainable results the regions must put their work within a larger system context. Previously, there has been performance based (national) initiatives, do this, fill quotas, measure x to get funded. Here more is about improvement of areas, based on regional gaps and prerequisites. Here the regions must ensure that they can identify their gaps and can communicate the reasons for their choices.” |
| **2018:** They are managers and must ask for results. Managers creates conditions for improvement work at the micro level. Our task is to find ways to urge them to create good conditions. The team can illuminate the gains and the gaps and ask questions like ‘what would the benefits be from working differently e.g., by including the patient perspective?’ If doing so, they may create a better work environment, facilitate recruitment, and improve the economy. Managers understand this. Through the strategic plan we have provided some tools as support, also when meeting managers at different levels during regional visits.” |
| **Observations 2021 Contact person meeting** *Discussion - activity report template.* 1) Limited possibility to report interventions for the area ‘women's health in general’; 2) Difficult to compare the already imple-mented with the planned interventions, despite overlap; 3) Difficult to provide some free-text answers; 4) 2021 supplementary agreement entails new accounting requirements, e.g., specifically reports of efforts. |
| ***7. Respond Rapidly*** |
| **Interviews, program team members**  **2020:** [effects of the pandemic] One conference was cancelled. In other ways, it has been positive. Contact persons meetings have improved: it has become easier to gather [in video meetings], you can have more frequent, but shorter, meetings, it does not have to be two-day sessions like before. Our digital workspace is used more frequently now. However, you must clarify more when you send information.” |
| **2018:** You must follow what happens in the world, so [strategies] change all the time. Neonatal care is added to the program, and the care chain strategy was not as clear from the beginning. Seeing the entire care process has emerged as important. In the beginning the politicians highlighted delivery care, now it is to develop the entire chain of care before, during and after childbirth. This is an example of strategy change. |
| **Archival data 2019** *Contact persons meeting, National Pregnancy Survey*: 1) What do we need to prepare together before the national launch; 2) Preparing an offer for the regions and time schedule; 3) Coordin-ation between key actors; 3) The Care guide platform as a channel; 4) Plan for exchanges of results? 5) Regional dissemination plans; 6) Preparing the message for different channels (program team develops) |
| **Observations 2021 – Program team meetings** *Discussion on women's health from a life cycle perspective:* "We have listened to many people's stories through our interviews [investigating work with the care chain]. Many regions or local actors work with parts of the chain, but the wholeness of the care process is missing. The purpose of our investigation is to show the gaps – how well does healthcare meet women's needs? This is good input to the Ministry and a good base for the regional work. New groups emerged, those who have performed genital surgeries, the LGBTQ group, and people with functional impairments" |
| ***8. Translate Findings*** |
| **Interviews, program team members**  **2020:** “I use the results [from activity reports, pregnancy registry] in dialogue meetings with the regions. First, we show national results, good and less good. Then the regional figures, good results and potentials for improvements. We notice an effect right away. People call us: “That indicator you showed, we have worked on it”. […] In our report, we use the structure of the strategic plan and from the dialogue meetings.” |
| **2020:** Our last summary report is a huge development. In our initial summary reports, we did not have the strategic plan. Now we use the reports more and tomorrow we will present the results to the Ministry. […] The more the program develops and the more tools we get, the more useful the reports become.” |
| **Observations 2021, program team meetings** *Discussion on communication activities:* “The Communications department is responsible for everything related to SALAR's communication. Competence is centralized to be able to coordinate. SALAR tries to be visible on social media, rankings are high in terms of facts and credibility, something to cherish and protect. Communication should be anchored, coordinated, and understandable. A manual for working with and visualizing the survey (e.g., PowerPoints, press-releases, social media messages) is ready - to help the regions with their external and internal communication.” |
